# Diagnostic Accuracy of Artificial Intelligence Applications on a Diverse Skin Image Set

**DOI:** 10.7759/cureus.102354

**Published:** 2026-01-26

**Authors:** Amiya K Shah, Megha Agarwal

**Affiliations:** 1 Chemistry, Viewpoint School, Calabasas, USA; 2 Cardiology, University of California Los Angeles, Los Angeles, USA

**Keywords:** applications, artificial intelligence, cancer, diagnosis, skin

## Abstract

Background

Several new mobile applications (apps) have been developed that utilize artificial intelligence (AI) to diagnose skin lesions.

Objective

The goal of this study was to evaluate the diagnostic accuracy of the most popular smartphone apps using a database of skin lesion images with diverse skin tones. An additional goal was to measure the apps’ sensitivity and specificity in detecting skin cancer.

Methods

A thorough search was performed in the Google Play Store and Apple App Store to find the most popular skin apps that diagnose skin lesions. We used the Stanford Diverse Dermatology Images database (DDI) to test the accuracy of the following apps: ChatGPT (OpenAI, San Francisco, CA, USA), AI skin scanner Rash Detector (by I Lov Guitars Inc., Scarborough, ON), Rash ID (Appsmiths LLC, Canton, MS USA), and Skin Scanner Dermatology & Acne (ACINA, UAB, located at Krokuvos, Vilnius, Lithuania). One hundred and two images with a range of diagnoses were selected for upload to each app. Fifty-one images were malignant, and 51 were benign. We also trained a new model of ChatGPT using a separate set of 554 images from the same database.

Results

All the apps had low diagnostic accuracy. The overall accuracy was 22%. When classifying benign versus malignant diagnoses, the apps had an average sensitivity of 46.57% and an average specificity of 72.06%. The average positive predictive value was 67.44%, and the average negative predictive value was 58.06%. In our study, training ChatGPT did not improve its diagnostic accuracy.

Conclusions

ChatGPT, Rash Detector, Rash ID, and Skin Scanner Dermatology & Acne performed poorly at diagnosing skin lesions from a database with diverse skin tones. These apps should not be used as stand-alone diagnostic tools.

## Introduction

Skin cancer is very common; approximately 9,500 people are diagnosed with skin cancer every day in the United States [1]. With increasingly limited access to dermatologists, especially in urban and rural areas, mobile applications (apps) that can diagnose skin lesions have the potential to help triage which patients need to consult a dermatologist [2].

Several mobile apps have been developed that utilize smartphone cameras and artificial intelligence (AI) to provide skin lesion diagnoses. These apps have mostly been trained using images that lack diversity in skin tone and are not FDA-approved [3,4]. The objective of this study was to evaluate the diagnostic accuracy of the most popular smartphone apps using a database of images with diverse skin tones. An additional goal was to measure the apps’ sensitivity and specificity in detecting skin cancer.

## Materials and methods

Database 

The database used for this study was the Diverse Dermatology Images (DDI) database published by Stanford University [5]. We selected this database because of its public availability and pathologically confirmed image dataset with diverse skin tones. In addition, the images are not meant to be textbook examples but rather represent the kind of clinical photos that AI algorithms may encounter in practice. This database was retrospectively created from pathology reports in Stanford clinics from 2010 to 2020. The database has 656 macroscopic images representing 570 unique patients. It does not have any dermoscopic images. The Fitzpatrick scale, established in 1975, was utilized to categorize the skin tone of each image based on in-person evaluation at the clinic visit, cross-referenced against demographic photos, and review of the clinical images by two board-certified dermatologists [6]. Each image is labeled with the skin tone (Fitzpatrick skin types: I-II, III-IV, V-VI). 

Selection of mobile apps 

A thorough search was performed in the Google Play Store and Apple App Store to find mobile apps that diagnose skin lesions. The search terms utilized were combinations of the following: skin, dermatology, lesion, condition, diagnosis, scanner, app, and artificial intelligence. The included apps all utilize AI to provide the diagnosis and allow uploading of skin images from a mobile device (Table 1). The following apps were used: ChatGPT (OpenAI, San Francisco, CA, USA), AI skin scanner Rash Detector (by I Lov Guitars Inc., Scarborough, ON), Rash ID (Appsmiths LLC, Canton, MS, USA), and Skin Scanner Dermatology & Acne (ACINA, UAB, located at Krokuvos, Vilnius, Lithuania). Apps that provided a remote consultation with a dermatologist were excluded from this study because this was considered similar to an in-person visit with a dermatologist and because the DDI database does not include symptoms or demographic data. Many of the apps found in the search that were excluded were related to skin care, not skin lesion diagnosis. The AI Dermatologist app was initially included but later excluded because the app generated error messages for the majority of uploaded images and thus could not be evaluated. 

**Table 1:** Applications that were tested

Mobile Application	Platform	Version Tested	User Rating	Cost
ChatGPT	Android, iOS	GPT- 4o	4.9 stars (iOS), 4.8 stars (Android)	Free
Rash Detector: Skin scanner	Android	4.0.3	1.0 star	$9.99 per week
Rash ID: AI skin scanner	iOS	4.0.1	3.3 stars	$5 per week
Skin Scanner Dermatology & Acne	iOS	2.0.2	4.1 stars	$6 per week

Testing the apps

A power analysis for an exploratory pilot study with a 75% confidence interval to estimate sensitivity with ±5% precision determined that a minimum of 48 malignant lesions would be needed. In our study, 102 images were randomly selected from the DDI Database with a range of diagnoses and equal distribution among skin types and benign/malignant lesions. Fifty-one images had the diagnosis of skin cancer, and 51 images were benign lesions. The images were equally distributed among the three skin type groups (Fitzpatrick skin types I-II, III-IV, and V-VI) [6]. The malignant diagnoses were melanoma, basal cell carcinoma, squamous cell carcinoma, mycosis fungoides, subcutaneous T-cell lymphoma, Kaposi sarcoma, metastatic carcinoma, and sebaceous carcinoma. The inflammatory skin conditions tested were pyogenic granuloma, keloid, and actinic keratosis.

**Figure 1:**
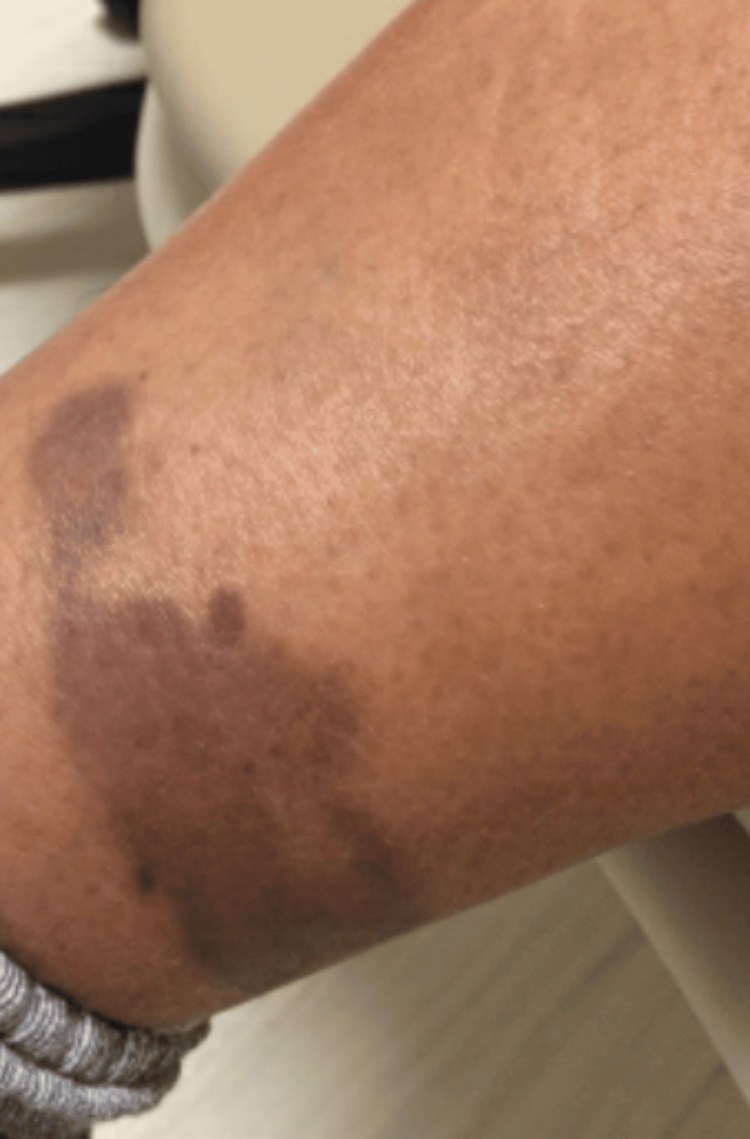
Original Image of Kaposi sarcoma from the Diverse Dermatology Images (DDI) Database, Fitzpatrick V-VI After analyzing this image, each of the applications tested provided the following diagnoses: ChatGPT:  post-inflammatory hyperpigmentation; Rash Detector: Skin Scanner, post-inflammatory hyperpigmentation; Rash ID: AI Skin Scanner, post-inflammatory hyperpigmentation; Skin Scanner Dermatology & Acne: ecchymosis; Trained GPT: acrochordon Note: De-identified clinical image sourced from the Diverse Dermatology Images (DDI) dataset (DOI: 10.71718/kqee-3z39), Stanford Center for Artificial Intelligence in Medicine & Imaging (AIMI).

All 102 images were individually uploaded into each app using the original files from the DDI Database. This was performed by a single operator (Amiya Shah), who was blinded to the image diagnosis. Images were uploaded and tested three times in each app for consistency. The portable network graphics (PNG) raster image file size from the DDI Database ranged from 110 KB to 610 KB. The diagnoses of the images used to test the apps included basal cell carcinoma, squamous cell carcinoma, melanoma, mycosis fungoidosis, subcutaneous lymphoma, Kaposi sarcoma, metastatic carcinoma, verruca vulgaris, epidermal nevus, epidermal cyst, pyogenic granuloma, seborrheic keratosis, neurofibroma, dermatofibroma, lipoma, acrochordon, keloid, melanocytic nevi, angioma, angioma, wart, onychomycosis, actinic keratosis, and solar lentigo (Figure 1). 

Training a new AI model 

To determine if ChatGPT's performance could be improved by training, we created a new AI GPT model using OpenAI ChatGPT tools. This model was trained using a separate set of images with their associated diagnoses. The training set consisted of the 554 images not used in our study to test the apps. OpenAI’s machine learning algorithm was instructed to use transfer learning and fine-tuning on the training dataset for the new GPT AI model. After training was completed, the new GPT AI model was then tested using the 102 images that were previously used to test the apps. 

## Results

All the applications performed poorly in making the correct diagnosis, with an average accuracy of 22.1% (Table 2). There was fair diagnostic agreement between applications (Fleiss’ Kappa = 0.278, p < 0.001). The applications performed the best at diagnosing squamous cell carcinoma but still had low accuracy (37%). The applications were least accurate at diagnosing melanocytic nevi (14.6% accuracy) and melanoma (23.4% accuracy). ChatGPT and Skin Scanner Dermatology & Acne performed the best, correctly diagnosing about one in four skin lesions. There was a statistically significant difference in diagnostic agreement between applications with regard to skin tone (Table 2). 

**Table 2:** Diagnostic accuracy across skin tones

	Fitzpatrick I–II, n (%)	Fitzpatrick III–IV, n (%)	Fitzpatrick V–VI, n (%)
All applications	34/136 (25.0)	30/136 (22.1)	25/136 (18.4)
ChatGPT	10/34 (29.4)	9/34 (26.5)	6/34 (17.7)
Rash Detector: Skin scanner	5/34 (14.7)	7/34 (20.6)	5/34 (14.7)
Rash ID: AI skin scanner	9/34 (26.5)	8/34 (23.5)	6/34 (17.7)
Skin Scanner Dermatology & Acne	10/34 (29.4)	6/34 (17.7)	8/34 (23.5)
Fleiss’ Kappa	0.038	0.430	0.396

**Figure 2:**
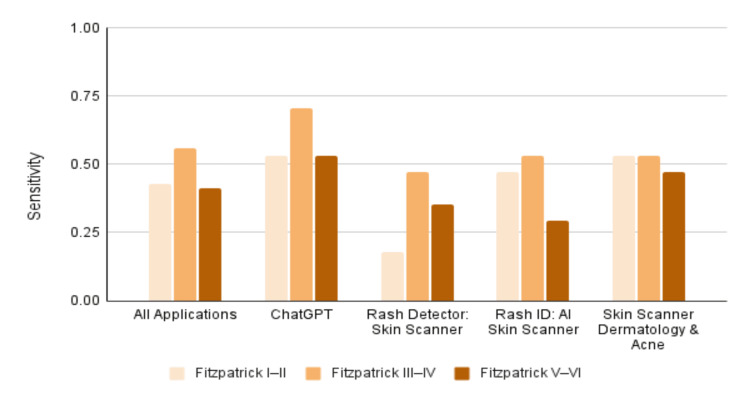
Sensitivity across skin tones Source: [6]

When distinguishing between benign versus malignant diagnoses, the average sensitivity was 46.6%, and the average specificity was 72.1% (Table 3). Generally, sensitivity varied across skin type groups (chi-square (χ²) = 3.59, p = 0.167; Table 4). The overall sensitivity for skin type I-II was 42.7%, skin type III-IV was 55.9%, and skin type V-VI was 41.2% (Figure 2).

**Table 3:** Applications' overall performance in detecting cancer

	Sensitivity	Specificity	F1	True positive	False negative	False positive	True negative
All applications	0.466	0.721	0.534	95	109	57	147
ChatGPT	0.588	0.804	0.659	30	21	10	41
Rash Detector: Skin scanner	0.333	0.824	0.442	17	34	9	42
Rash ID: AI skin scanner	0.431	0.647	0.484	22	29	18	33
Skin Scanner: Dermatology & Acne	0.510	0.608	0.536	26	25	20	31

**Table 4:** Applications' performance in detecting cancer across skin tones

	Fitzpatrick I–II		Fitzpatrick III–IV		Fitzpatrick V–VI	
	Sensitivity	Specificity	Sensitivity	Specificity	Sensitivity	Specificity
All applications	0.427	0.794	0.559	0.677	0.412	0.691
ChatGPT	0.529	0.882	0.706	0.765	0.529	0.765
Rash Detector: Skin Scanner	0.177	0.100	0.471	0.706	0.353	0.765
Rash ID: AI Skin Scanner	0.471	0.647	0.529	0.647	0.294	0.647
Skin Scanner Dermatology & Acne	0.529	0.647	0.529	0.588	0.471	0.588

Melanoma was commonly missed by all applications, with a 62.5% miss rate. The newly created and trained GPT model did not perform better than ChatGPT in sensitivity, specificity, or diagnostic accuracy.

## Discussion

The diagnostic accuracy of all the apps was poor. In the literature, the diagnostic accuracy of AI-based apps varies widely, ranging from 22% to 81% [7-11]. Our overall diagnostic accuracy was similar to the 22.8% reported accuracy of the Skin Image Search AI app [8]. However, this app is no longer available. In our study, the apps’ average sensitivity was 46.6%, which is clinically unacceptable for cancer screening.

We only found two studies that reported fairly high diagnostic accuracy. A 2024 study assessing ChatGPT found an overall accuracy of 81.2% [9]. This study focused on 17 dermatologic conditions, comparing the light skin tone and darker skin tone groups. However, diagnoses with similar clinical appearances were not tested in this study. For example, ten images of melanoma were tested in the study, but no images of melanocytic nevi were included. This could explain the relatively high reported diagnostic accuracy. In contrast, our study used 32 different diagnoses, many with similar clinical appearances. The second study with a fairly high diagnostic accuracy was a 2023 study by Marri et al. that found an 80.5% accuracy of the Tibot app [7]. However, this study used only 12 diagnoses and did not test different skin tones. In addition, some diagnostic categories were described as nonspecific classifications, such as “suspicious tumors”. The Tibot app is no longer available. 

Aysa is a popular app on iOS and Android platforms that provides a probable diagnosis based on clinical images, skin type, patient demographics, and associated symptoms. A 2024 clinical study of this app in Asian patients with Fitzpatrick skin types III-V found a wide range of diagnostic performance [10]. The F1 score for bacterial infections was 0.857, but the F1 score for malignant tumors was 0.173. Marri et al. concluded that the app had promising outcomes for some issues, such as infections and inflammatory disorders, but was unreliable at detecting malignant tumors [7]. 

One reported concern regarding mobile AI dermatology apps is skin tone bias [3]. The issue is that the images used to train the AI models are generally of lighter skin tones and are not representative of the diverse skin tones of the general population. Skin tone bias was found in a 2025 study evaluating ChatGPT, where the diagnostic accuracy decreased by 20% for individuals with skin of color [9]. Daneshjou et al. tested three different AI algorithms on the DDI database [11]. They also found a bias regarding skin tone. Receiver operating characteristic-area under the curve (ROC-AUC) performance was better on the subset of Fitzpatrick I-II compared to Fitzpatrick V-VI images, with ModelDerm having an ROC-AUC of 0.64 versus 0.55, DeepDerm having an ROC-AUC of 0.61 versus 0.50, and HAM10000 having an ROC-AUC of 0.72 versus 0.57. In our study, we observed variation in skin cancer sensitivity and specificity by Fitzpatrick skin type. 

Skin tone can affect the macroscopic and dermoscopic appearance of skin diseases [12]. In fact, due to the difficulty of recognizing erythema in darker skin tones, the severity of the respective inflammatory diseases in these individuals is often underestimated and can lead to bias in AI models [13, 14]. Skin cancer is less common in people with darker skin tones but is often associated with greater morbidity and mortality [15]. It is important for AI tools to be trained on these lesions to maximize the likelihood of early skin cancer detection in people with darker skin tones. One barrier is the underrepresentation of these lesions; Fitzpatrick V-VI skin tone images constituted only 10.5% of all skin images in medical textbooks [16]. 

We expected the new GPT model trained with images from the same DDI database to outperform ChatGPT, but surprisingly, it did not. This could be because the training dataset was not large enough and because large language models like ChatGPT were not designed to analyze images. To improve accuracy, future AI-based apps may need to use a different approach, like the convolutional neural network (CNN) technology. 

CNN is a type of deep learning algorithm that includes a convolutional layer, a pooling layer, and a fully connected layer [17]. Convolutional and pooling layers extract features from the lesion and reduce computational power, whereas fully connected layers classify the image into two or more categories. CNN is better adapted for interpreting images because it mimics the human visual cortex. CNN has been developed to detect melanoma and classify malignant lip diseases [17-19]. One algorithm was shown to assist medical professionals in diagnosing skin cancer by 83.8% [20]. Other studies have also shown CNN technology to assist general practitioners in diagnosing skin lesions [21, 22]. Ba et al. reported that CNN-assisted dermatologists achieved significantly higher diagnostic accuracy (76.60% vs. 62.78%), especially dermatologists with less experience [23]. For specific scenarios, AI software has been shown to perform as well as or better than dermatologists. For example, a systematic review in Cancers found that AI algorithms using dermoscopic images were more accurate than dermatologists in the detection of melanoma [24]. 

In our study, the mobile apps we tested did not meet the necessary standards for a stand-alone smartphone diagnostic tool. A recent JAMA review of dermatology mobile apps came to the same conclusion [25]. Currently, mobile AI software is not accurate enough to replace a consultation with a dermatologist, but it does have the most potential to assist clinicians in correctly diagnosing difficult-to-classify skin lesions. A recent systematic review concluded that AI has the most potential to assist clinicians, especially non-dermatologists, in improving the overall accuracy of skin cancer diagnosis [26]. 

The International Skin Imaging Collaboration Artificial Intelligence Working Group published consensus guidelines in 2022 for all aspects of AI development in dermatology [27]. The recommendations are grouped into the topics of data, technique, assessment, and app. Guidelines were created as a checklist to address current challenges in dermatology image-based AI that impair clinical translation, including a lack of image standardization, concerns about potential sources of bias, and factors behind performance degradation. Ethical considerations and potential sources of bias unique to clinical photography were also addressed. These recommendations should be considered for future AI developments in dermatology. 

Limitations

A limitation of our study was the utilization of one database to assess app performance. The DDI database was the only publicly available database that included Fitzpatrick skin types. As more databases with diverse skin tones become available, it will be interesting to see how the latest mobile apps perform across several databases. Also, the test set of 102 images was relatively small. 

Another limitation was the lack of demographic data and symptoms in the DDI database. The DDI database only consists of clinical images, diagnoses, and Fitzpatrick skin types. We were unable to test the popular Aysa app because it requires age, demographic information, signs, and symptoms, along with an image of the skin lesion to provide a differential diagnosis to the user. We were also unable to test the AI Dermatologist app because it generated error messages for the vast majority of uploaded images. 

## Conclusions

ChatGPT, Rash Detector, Rash ID, and Skin Scanner Dermatology & Acne had poor sensitivity and specificity for skin cancer from a database with diverse skin tones. Additionally, these mobile applications performed poorly at diagnosing skin lesions. Based on current performance, none of the applications we tested meet the necessary standards for a stand-alone smartphone diagnostic tool. Creating more databases with diverse skin images, using CNNs, and seeking input from dermatologists in algorithm development should help mobile AI apps meet the necessary standards in the future.
